# Retrospective study of *RAS/PIK3CA/BRAF* tumor mutations as predictors of response to first-line chemotherapy with bevacizumab in metastatic colorectal cancer patients

**DOI:** 10.1186/s12885-016-2994-6

**Published:** 2017-01-09

**Authors:** Izuma Nakayama, Eiji Shinozaki, Tomohiro Matsushima, Takeru Wakatsuki, Mariko Ogura, Takashi Ichimura, Masato Ozaka, Daisuke Takahari, Mitsukuni Suenaga, Keisho Chin, Nobuyuki Mizunuma, Kensei Yamaguchi

**Affiliations:** Department of Gastroenterology, Cancer Institute Hospital of the Japanese Foundation for Cancer Research, 3-8-31 Ariake, Koto-ku, Tokyo, 135-8550 Japan

**Keywords:** *RAS* mutation, *PIK3CA* mutation, *BRAF* mutation, Colorectal cancer, bevacizumab

## Abstract

**Background:**

After analysis of minor *RAS* mutations (*KRAS* exon 3, 4/*NRAS*) in the FIRE-3 and PRIME studies, an expanded range of *RAS* mutations were established as a negative predictive marker for the efficacy of anti-EGFR antibody treatment. *BRAF* and *PIK3CA* mutations may be candidate biomarkers for anti-EGFR targeted therapies. However, it remains unknown whether *RAS/PIK3CA/BRAF* tumor mutations can predict the efficacy of bevacizumab in metastatic colorectal cancer. We assessed whether selection according to *RAS/PIK3CA/BRAF* mutational status could be beneficial for patients treated with bevacizumab as first-line treatment for metastatic colorectal cancer.

**Methods:**

Of the 1001 consecutive colorectal cancer patients examined for *RAS, PIK3CA*, and *BRAF* tumor mutations using a multiplex kit (Luminex®), we studied 90 patients who received combination chemotherapy with bevacizumab as first-line treatment for metastatic colorectal cancer. The objective response rate (ORR) and progression-free survival (PFS) were evaluated according to mutational status.

**Results:**

The ORR was higher among patients with wild-type tumors (64.3%) compared to those with tumors that were only wild type with respect to *KRAS* exon 2 (54.8%), and the differences in ORR between patients with wild-type and mutant-type tumors were greater when considering only *KRAS* exon 2 mutations (6.8%) rather than *RAS/PIK3CA/BRAF* mutations (18.4%). There were no statistically significant differences in ORR or PFS between all wild-type tumors and tumors carrying any of the mutations. Multivariate analysis revealed that liver metastasis and *RAS* and *BRAF* mutations were independent negative factors for disease progression after first-line treatment with bevacizumab.

**Conclusions:**

Patient selection according to *RAS/PIK3CA/BRAF* mutations could help select patients who will achieve a better response to bevacizumab treatment. We found no clinical benefit of restricting combination therapy with bevacizumab for metastatic colorectal cancer patients with EGFR-wild type tumors.

**Electronic supplementary material:**

The online version of this article (doi:10.1186/s12885-016-2994-6) contains supplementary material, which is available to authorized users.

## Background

The EGFR signaling pathway has a key role in the proliferation and survival of colorectal cancer cells. Point mutations in exon 2 of the *KRAS* gene have been shown to be negative predictive markers of the response to anti-EGFR treatment, and consequently anti-EGFR antibodies were not administered to patients with *KRAS* exon 2 mutant tumors [1]. After a retrospective analysis of minor *RAS* mutations (e.g. *KRAS* exon 3 and 4/*NRAS*) in the FIRE-3 and PRIME studies [2, 3], the so called “all *RAS* mutation” also came to be regarded as a negative biomarker for anti-EGFR antibody treatment [4]. In addition to *RAS*, *BRAF* and *PIK3CA* mutations are potential biomarkers of response to anti-EGFR targeted therapies [5]. However, it remains unknown whether EGFR pathway mutations affect the efficacy of bevacizumab (Bmab) in metastatic colorectal cancer (mCRC). We evaluated the significance of *RAS/PIK3CA/BRAF* tumor mutations in patients receiving combination chemotherapy with Bmab as the first-line treatment for mCRC, and we assessed whether these mutations could be used to select patients who would derive the greatest clinical benefit from Bmab.

## Methods

### Patients

This was a retrospective study conducted at a single Japanese institute and approved by the ethics committee of Cancer Institute Hospital of Japanese Foundation for Cancer Research (No.2009-1048). Of the 1001 consecutive patients with histologically confirmed CRC who were examined for tumor *RAS, PIK3CA*, and *BRAF* mutations in our institute between November 2006 and December 2013, 90 patients were administered combination chemotherapy with Bmab as the first-line treatment for mCRC. Patients who received neo-adjuvant chemotherapy (NAC) or adjuvant chemotherapy completed less than 6 months before enrollment to this study were excluded. Patients who had undergone surgery for metastatic sites were included if it had been performed more than 4 weeks earlier. Patients were required to have adequate hematologic, hepatic, cardiac, and renal function. Their medical records were reviewed to obtain data on clinicopathologic variables. All patients provided written informed consent before receiving treatment.

### Procedure

The treatment regimen was determined by the physician for each patient. The following regimens were employed: modified FOLFOX6 plus Bmab consisted of a fortnightly course of Bmab (5 mg/kg intravenously over 30 to 90 min on day 1), oxaliplatin (85 mg/m^2^ intravenously over 2 h on day 1) plus l-LV (200 mg/m^2^ intravenously over 2 h on day 1) and 5-fluorouracil (5-FU) (400 mg/m^2^ bolus on day 1, followed by infusion of 2400 mg/m^2^ over 46 h); and CapeOX plus Bmab consisted of oxaliplatin (130 mg/m^2^ intravenously over 2 h on day 1) plus oral capecitabine (1000 mg/m^2^ twice daily for 2 weeks in a 3-week cycle). Bmab (7.5 mg/kg) was administered ahead of oxaliplatin intravenously on day 1 every 3 weeks. FOLFIRI plus Bmab consisted of fortnightly courses of Bmab (5 mg/kg intravenously over 30 to 90 min on day 1), irinotecan (150 mg/m^2^ intravenously over 2 h on day 1) plus l-LV (200 mg/m^2^ intravenously over 2 h on day 1) and 5-FU (400 mg/m^2^ bolus on day 1, followed by infusion of 2400 mg/m^2^ over 46 h).

DNA was extracted from formalin-fixed paraffin-embedded (FFPE) tumor tissue, which was mostly obtained at biopsy. Mutations in *KRAS* codons 12 and 13 were examined using a kit based on a Luminex assay (MEBGEN *KRAS* Mutation Detection kit, MBL). A Luminex based kit (GENOSEARCH Mu-PACK, MBL) was also used to detect a total of 36 mutations in *KRAS* (codons 61 and 146), *NRAS* (codons 12, 13, and 61), *PIK3CA* (codons 542, 545, 546, and 1047) and *BRAF* (codon 600). The concordance of findings based on this newly developed multiplex assay kit with conventional direct sequencing results was confirmed previously [6].

### Statistical analysis

The objective response rate (ORR) was evaluated according to the Response Evaluation Criteria in Solid Tumors (RECIST) ver. 1.1. The progression-free survival (PFS) and overall survival (OS) were calculated using the Kaplan-Meier method. PFS was defined as the duration of survival from the start of chemotherapy to the date of recurrence or death from any cause, whichever occurred first. Patients with no recurrence until the cut-off date were regarded as censored on the last date when no recurrence had been proven by imaging. The disease-progression date was retrospectively re-analyzed by the investigator, and was defined as the date on which progression was first detected using a computed tomography (CT) or fluorodeoxyglucose-positron emission tomography (FDG-PET) scan. If treatments were discontinued before or continued after disease-progression due to adverse events or the patient’s request, they were censored at the time of the last radiological examination. OS was defined as survival from the start of chemotherapy to death from any cause. For patients who were lost to follow-up, data were censored on the date when the patient was last known to be alive. The data cut-off date was August 12, 2015. A one-sided Fisher’s exact test was used to assess the statistical significance of the difference between ORRs according to mutational status at a significance level of 2.5%. Both PFS and OS were estimated using the Kaplan-Meier method and compared using the log-rank test at a significance level of 5%. In addition to *RAS*, *PIK3CA*, and *BRAF* tumor mutations, variables with a *P* value less than 0.05 in a univariate analysis were included in a multivariate Cox regression analysis. All analyses were performed with EZR (Saitama Medical Center, Jichi Medical University, Saitama, Japan), which is a graphical user interface for R software (The R Foundation for Statistical Computing) [7].

## Results

### Baseline characteristics

The baseline characteristics of the patients are shown in Table 1. Their median age was 63 years (range, 27–79 years). Forty-eight patients (53.3%) were men and 42 patients (46.7%) were women. Almost all of the subjects had a good performance status. Seventy-four patients (82.2%) had colon cancer and 16 (17.8%) had rectal cancer, including right-sided colon cancer (RCC) in 34 patients (37.8%) and left-sided colorectal cancer (LCRC) in 56 patients (62.2%). RCC was defined as a tumor arising from the cecum to the transverse colon, excluding the appendix, while LCRC was defined as a tumor arising from the descending colon to the rectum. Of these 90 patients, the tumors of 43 patients (46.7%) were found to have a mutation in *KRAS* exon 2. In total 48 patients (53.3%) had a *RAS* mutation (*KRAS/NRAS*). Seven patients (8.9%) had a *PIK3CA* mutation, and another 7 (8.9%) had a *BRAF* mutation. Thirty-three patients (36.7%) had tumors with no *RAS, PIK3CA*, or *BRAF* mutation.

**Table 1:** Baseline patient characteristics (*n* = 90)

Characteristic	N (%)
Sex	
Male	48 (53.3)
Female	42 (46.7)
Median age (range), years	63 (27–79)
ECOG performance status	
0	82 (91.1)
1, 2	8 (8.9)
Site of primary tumor	
RCC (cecum to the transverse colon)	34 (37.8)
LCRC (descending to the rectosigmoid colon)	40 (44.4)
Rectum	16 (17.8)
Mode of metastasis	
Synchronous	77 (85.6)
Asynchronous	13 (14.4)
Sites of metastasis	
Liver	51
Lung	38
Distant lymph nodes	29
Peritoneum	23
Histology	
Differentiated	83 (92.2)
Undifferentiated	7 (7.8)
Number of metastases	
1	37 (41.1)
≥ 2	53 (58.9)
Chemotherapy regimen	
FOLFOX4/mFOLFOX6 or XELOX	86 (95.6)
FOLFIRI	4 (4.4)
Prior metastatectomy	
Yes	12 (13.3)
No	78 (86.7)
Resection of primary tumor	
Yes	67 (74.4)
No	23 (25.6)
Previous oxaliplatin treatment as adjuvant CTx	
Yes	13 (14.4)
No	77 (85.6)
*KRAS* status (codon 12,13)	
Wild-type	47 (52.2)
Mutant	43 (47.8)
*RAS* status (*KRAS/NRAS*)	
Wild-type	42 (46.7)
Mutant	48 (53.3)
*PIK3CA* status	
Wild-type	82 (91.1)
Mutant	8 (8.9)
*BRAF* status	
Wild-type	82 (91.1)
Mutant	8 (8.9)

*ECOG* Eastern Cooperative Oncology Group, *RCC* right-sided colon cancer, *LCRC* left-sided colorectal cancer, *CTx* chemotherapy

### Treatment exposure

Almost all patients received an oxaliplatin-containing regimen, which was FOLFOX in 34 cases (37.8%) and XELOX in 52 cases (57.8%). Among them, 13 (14.4%) had been administered oxaliplatin prior to this treatment as an adjuvant therapy. The primary tumor was resected in 67 patients (74.4%) and 13 patients (14.4%) underwent a metastatectomy.

### ORR, PFS, and OS

**Fig. 1:**
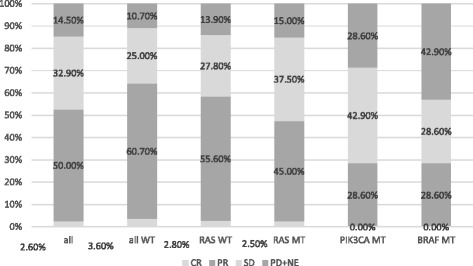
Response according to tumor mutation status

**Fig. 2:**
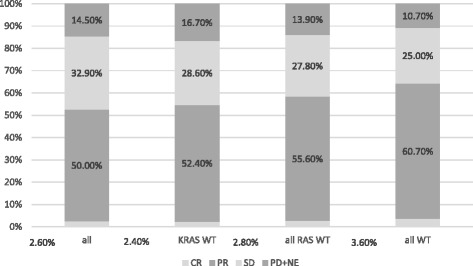
Responses among patients with wild-type tumor *KRAS* exon 2, *RAS*, and *RAS/PIK3CA/BRAF*

The median follow-up period for all eligible patients was 23.5 (0.8–41.4) months, and 51 patients (56.7%) died by the cut-off date. Seventy-seven of the 90 patients had measurable lesions. The overall ORR was 52.6% whilst the ORR of patients with no detected tumor mutations was 64.3%. The ORRs of patients with a *PIK3CA* or *BRAF* tumor mutation were very low (28.6%), and more than 40% of patients with a *BRAF* tumor mutation had confirmed disease progression at the first evaluation (Fig. 1). Although the ORR varied according to the mutational status of the tumor, these differences were not statistically significant (Table 2). The differences in ORR became gradually greater among patients with wild-type tumors as restricting treatment subjects from only wild type with respect to *KRAS* exon 2, all *RAS* wild-type toward all wild-type (*RAS/PIK3CA/BRAF*). The difference in ORR between the whole population and patients with all wild-type tumors was 11.7% (Fig. 2).

**Table 2:** Response according to mutational status

	*KRAS* exon 2 wt vs. mt	*RAS* wt vs. mt	*PIK3CA/BRAF* wt vs. mt	*RAS*/*PIK3CA*/*BRAF* All wt vs. any mt
Responder (n)	23 vs. 17	21 vs. 19	38 vs. 2	18 vs. 22
Non-responder (n)	19 vs. 18	15 vs. 21	31 vs. 5	10 vs. 26
ORR (%)	54.8 vs. 48.6	58.4 vs. 47.5	55.1 vs. 28.6	64.3 vs. 45.9
Difference in ORR	6.8	10.9	26.5	18.4
*P* value*	0.651	0.368	0.246	0.155

Responder, CR + PR; Non-responder, SD + PD + NE; ORR, overall response rate; wt, wild-type; mt, mutant

*calculated using Fisher’s exact test

**Fig. 3:**
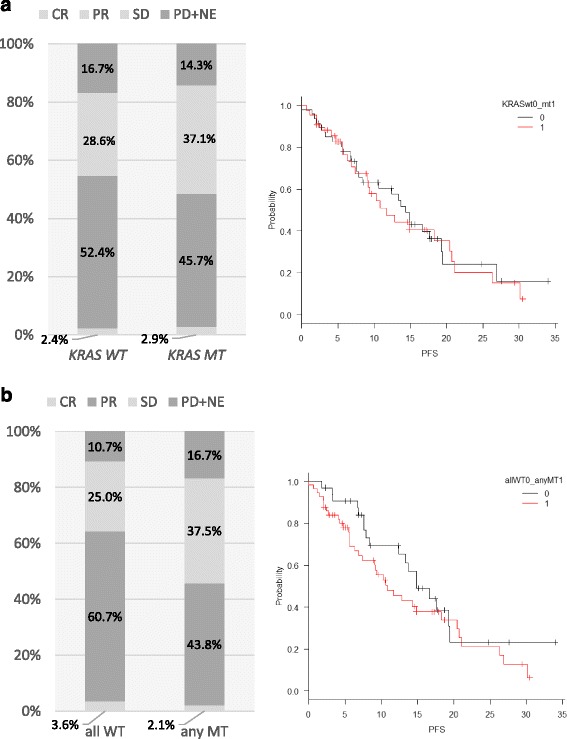
Relationship between overall response rate (ORR) and progression free survival (PFS) in patients with wild-type or mutant (**a**) *KRAS* exon 2 and (**b**) *RAS/PIK3CA/BRAF* tumors

The overall median OS and PFS were 27.7 and 13.3 months, respectively. The ORR of patients with *KRAS* exon 2 wild-type and mutant-type tumors differed by 6.2%, and these populations had nearly identical Kaplan-Meier curves for PFS (Fig. 3). The difference between ORRs (18.4%) was larger when comparing patients with wild-type tumors to those with a tumor carrying any mutation. There was no statistically significant difference in PFS between these groups, although there was a slightly larger difference in Kaplan-Meier curves (Fig. 3).

Univariate analysis revealed that an elevated serum C-reactive protein (CRP) level (>0.05 mg/dl), an unresectable primary tumor, and liver metastases were associated with a significantly shorter PFS (Table 3). Multivariate analysis that included *RAS, PIK3CA,* and *BRAF* tumor mutations and baseline prognostic variables revealed that liver metastasis, unresectable primary tumor, *RAS* and *BRAF* tumor mutations had independent prognostic value for early progression (Table 3).

**Table 3:** Univariate and multivariate analysis of pr4gression-free survival (PFS) (*n* = 90)

Variable	Univariate analysis		Multivariate analysis	
	HR (95% CI)	*P* value	HR (95% CI)	*P* value
Sex				
Male vs. female	1.0 vs. 0.72 (0.42–1.24)	0.236		
Age				
< 63 y.o vs. ≥63 y.o	1.0 vs. 0.82 (0.48–1.40)	0.472		
ECOG PS				
0 vs. 1,2	1.0 vs. 0.66 (0.21–2.14)	0.490		
Site of primary tumor				
RCC vs. LCRC	1.0 vs. 0.88 (0.51–1.52)	0.643		
Differentiated-type				
yes vs. no	1.0 vs. 1.78 (0.75–4.22)	0.188		
Synchronous mets				
yes vs. no	1.0 vs. 0.61 (0.29–1.30)	0.201		
Sites of metastasis				
Non-liver vs. liver	1.0 vs. 1.84 (1.06–3.20)	*0.031*	1.0 vs. 3.26 (1.57–6.77)	*0.002*
Non-lung vs. lung	1.0 vs. 1.17 (0.68–2.00)	0.567		
Non-LN vs. LN	1.0 vs. 0.67 (0.37–1.20)	0.177		
Non-P vs. P	1.0 vs. 0.95 (0.51–1.78)	0.878		
Number of metastases				
1 vs. ≥2	1.0 vs. 0.87 (0.51–1.50)	0.628		
Primary resection				
yes vs. no	1.0 vs. 2.00 (1.09–3.66)	*0.024*	1.0 vs. 2.13 (1.05–4.29)	*0.035*
Prior L-OHP				
yes vs. no	1.0 vs. 0.75 (0.35–1.60)	0.457		
ALP (/)				
≤ ULN vs. >ULN	1.0 vs. 1.07 (0.56–2.04)	0.836		
LDH (/IU)				
≤ ULN vs. >ULN	1.0 vs. 1.27 (0.74–2.21)	0.387		
CRP (mg/dl)				
≤ ULN vs. >ULN	1.0 vs. 2.35 (1.32–4.17)	*<0.001*	1.0 vs. 1.57 (0.79–3.10)	0.196
CEA				
≤ ULN vs. >ULN	1.0 vs. 0.90 (0.49–1.65)	0.735		
CA19-9				
≤ ULN vs. >ULN	1.0 vs. 1.63 (0.95–2.80)	0.076		
*RAS* status				
Wild-type vs. mutant	1.0 vs. 1.36 (0.79–2.32)	0.264	1.0 vs. 2.01 (1.07–3.76)	*0.030*
*PIK3CA* status				
Wild-type vs. mutant	1.0 vs. 1.00 (0.36–2.77)	0.993	1.0 vs. 0.66 (0.21–2.03)	0.466
*BRAF* status				
Wild-type vs. mutant	1.0 vs. 1.60 (0.68–3.75)	0.285	1.0 vs. 3.87 (1.38–10.9)	*0.010*

*CI* confidence interval, *HR* hazard ratio, *RCC* right-sided colon cancer, *LCRC* left-sided colorectal cancer, *mets* metastasis, *LN* lymph node, *P* peritoneum, *ULN* upper limit of normal, *NA* not assessable. All data in italics are with *p*-value <0.05

## Discussion

In clinical practice, it is often not as easy to conduct CT scans at regular intervals as it is in clinical trials. We considered that the ORR would be a relatively rigid parameter for evaluating the efficacy of first-line treatment in clinical practice. In this study, treatment was discontinued due to not only disease progression, but also adverse events or patient refusal, and 21 patients discontinued treatment before disease progression was confirmed by imaging. Time to treatment failure (TTF) is sometimes chosen as an endpoint instead of PFS in clinical trials. However, we considered that PFS would be a more suitable endpoint to evaluate the biological activity of the tumor and drug resistance compared to TTF. OS was the most rigid endpoint but would be determined by not only the first-line treatment but also by second-line and subsequent treatments. The difference in OS may be a result of anti-EGFR therapy after the first-line treatment in patients who have *KRAS* or *RAS* wild-type tumors. We therefore assessed the relationship between clinicopathologic factors including *RAS*, *PIK3CA*, and *BRAF* tumor mutation status and PFS.

In this study, we found that patients with a *RAS*, *PIK3CA*, or *BRAF* tumor mutation had a lower ORR than patients with tumors that did not carry these mutations, although this difference was not statistically significant. Patient selection according to tumor mutations in the EGFR pathway might improve the overall response to combination therapy with Bmab as a first-line treatment for mCRC. However, these differences in ORRs could not translate into an improved PFS. Multivariate analysis revealed a negative predictive value of *RAS* and *BRAF* tumor mutations with respect to first-line Bmab treatment. In this study, all patients were treated with Bmab, and hence, we could not clarify whether these gene mutations had predictive or prognostic value.

Data from preclinical research has indicated that changes in the EGFR signaling pathway might be related to the efficacy of anti-VEGF therapy [8]. Post-analysis of the AVF2107g trial revealed that adding Bmab to cytotoxic chemotherapy was beneficial regardless of *KRAS* exon 2 mutation status [9]. *KRAS* exon 2 mutations are not regarded as predictive markers of Bmab treatment. However, we found that the ORR, PFS, and OS differed between patients with *KRAS* wild-type and mutant tumors (ORR, 60.0% vs. 41.2%; PFS, 13.5 months vs. 9.3 months; OS, 27.7 months vs. 19.9 months). These findings are mostly similar to those of other trials comparing clinical outcomes between patients with *KRAS* exon 2 wild-type and mutant tumors [10–12]. Although statistically significant differences in OS and PFS between patients with *KRAS* exon 2 wild-type and mutant-type tumors were only shown in the MACRO trial [12], there were numerical differences shown all other trials. *KRAS* exon 2 wild-type tumors may predict a favorable prognosis. In a retrospective analysis of the PEAK, FIRE-3, and CALGB/SWOG80405 trials, this trend was also apparent when including subjects with any *RAS* tumor mutation, rather than just those with *KRAS* exon 2 tumor mutations [13–15]. A recent retrospective analysis of data from the TRIBE trial suggested that tumor mutations in both *BRAF* and *RAS* genes predicted a poor outcome for patients undergoing first-line treatment with Bmab plus FOLFIRI or FOLFOXIRI, although *RAS* mutations had less impact than *BRAF* mutations [16]. Larger patient numbers would be needed to translate the difference in ORR between patients with wild-type and mutant tumors into an improved clinical outcome, which may be why only the MACRO trial, with the largest number of patients, revealed statistically significant differences in outcome between patients with *KRAS* wild-type and mutant-type tumors.

Mutations in the *BRAF* gene have been shown to be markers of a poor prognosis following mCRC treatment [17, 18] and have a stronger prognostic value than *RAS* mutations [19]. However, *BRAF* mutant cases were very rare, only 8 in this cohort. It is therefore very difficult to obtain an adequate number of these cases to show statistically significant differences.

Taking into account these previous data, *RAS* and *BRAF* mutations may be associated with the inferior efficacy of Bmab treatment. However, due to the relatively small effect of *RAS* mutations and the rarity of *BRAF* mutations, we were unable to show statistically significant differences in the ORR and PFS of patients undergoing first-line treatment with Bmab for mCRC.

The ORR and PFS in patients with any of the examined mutations were 45.9% and 10.8 months, respectively, in this study. These were comparable with those of patients with *KRAS* exon 2 mutant tumors treated using FOLFOX4 alone in the OPUS study (ORR, 52.0%; PFS, 8.6 months) [20]. However, in the cetuximab arm of the OPUS and CRYSTAL trials, the ORR and PFS of patients with *KRAS* mutant tumors were much worse (ORR, 26.0 and 31.3% respectively; PFS, 5.5 and 7.4 months, respectively) than patients with no tumor mutations in our study [20, 21]. Our study did not show that mCRC patients with a tumor mutation in *RAS*, *PIK3CA*, or *BRAF* had a poorer response to combination chemotherapy with Bmab compared with patients who had none of these mutations. As such, there are insufficient data to justify the exclusion of patients with a *RAS*, *PIK3CA*, or *BRAF* tumor mutation from Bmab treatment regimens.

Our study has several limitations. Firstly, this was retrospective cohort study conducted at a single institute. Secondly, there was selection bias in the treatment of patients with *KRAS* wild-type tumors, especially those who had metastases only in the liver. In our institute, patients with *KRAS* wild-type tumors and liver only metastases were administered anti-EGFR therapy as an initial standard treatment in order to achieve conversion to metastatectomy. Patients with *KRAS* wild-type tumors and liver metastases in this cohort had relatively unfavorable factors, such as a high tumor burden or multiple organ metastases, and patients with relatively favorable factors were usually excluded. This may explain why liver metastasis was an independent predictor of a poor prognosis in this cohort. Patients with *RAS* wild-type tumors in this cohort might have had a poor prognosis, compared to those included in other studies, and this selection bias might have affected the outcome of patients with tumors that do not carry mutations in the genes studied here. Thirdly, due to the rare incidence of *PIK3CA* and *BRAF* mutation in CRC, we could evaluate only small number patients with these mutations.

## Conclusion

There were no statistically significant differences in ORR and PFS according to mutations in EGFR pathway genes in patients receiving cytotoxic chemotherapy with Bmab as the first-line treatment for mCRC. *RAS/PIK3CA/BRAF* mutations could help identify tumors that will respond to both anti-EGFR antibodies and Bmab. However, this study did not find a clinical benefit for restricting Bmab treatment to mCRC patients with tumors that have wild-type EGFR pathway genes.

## Additional file

Additional file 1: Row data about clinicopathological features and clinical outcomes with omitting personally identifiable information of patients. (XLSX 27 kb)

## Abbreviations

5-FU: 5-fluorouracil
Bmab: bevacizumab
CT: Computed tomography
EGFR: Epidermal growth factor receptor
FFPE: Formalin-fixed paraffin-embedded
LCRC: Left-sided colon
mCRC: Metastatic colorectal cancer
NAC: Neo-adjuvant chemotherapy
ORR: Objective response rate
OS: Overall survival
PET: Positron emission tomography
PFS: Progression free survival
RCC: Right-sided colon
RECIST: Response Evaluation Criteria in Solid Tumors
TTF: Time to treatment failure
